# Protein-tyrosine phosphorylation in *Bacillus subtilis*: a 10-year retrospective

**DOI:** 10.3389/fmicb.2015.00018

**Published:** 2015-01-23

**Authors:** Ivan Mijakovic, Josef Deutscher

**Affiliations:** ^1^Systems and Synthetic Biology, Department of Chemical and Biological Engineering, Chalmers University of Technology, Göteborg, Sweden; ^2^Centre National de la Recherche Scientifique, FRE3630 Expression Génétique Microbienne, Institut de Biologie Physico-Chimique, Paris, France; ^3^UMR1319 Microbiologie de l’Alimentation au Service de la Santé Humaine, Institut National de la Recherche Agronomique/AgroParisTech, Jouy en Josas, France

**Keywords:** protein phosphorylation, BY-kinase, phosphotyrosine-protein phosphatase, regulatory network, substrate specificity

## Abstract

The discovery of tyrosine-phosphorylated proteins in *Bacillus subtilis* in the year 2003 was followed by a decade of intensive research activity. Here we provide an overview of the lessons learned in that period. While the number of characterized kinases and phosphatases involved in reversible protein-tyrosine phosphorylation in *B. subtilis* has remained essentially unchanged, the number of proteins known to be targeted by this post-translational modification has increased dramatically. This is mainly due to phosphoproteomics and interactomics studies, which were instrumental in identifying new tyrosine-phosphorylated proteins. Despite their structural similarity, the two *B. subtilis* protein-tyrosine kinases (BY-kinases), PtkA and PtkB (EpsB), seem to accomplish different functions in the cell. The PtkB is encoded by a large operon involved in exopolysaccharide production, and its main role appears to be the control of this process. The PtkA seems to have a more complex role; it phosphorylates and regulates a large number of proteins involved in the DNA, fatty acid and carbon metabolism and engages in physical interaction with other types of kinases (Ser/Thr kinases), leading to mutual phosphorylation. PtkA also seems to respond to several activator proteins, which direct its activity toward different substrates. In that respect PtkA seems to function as a highly connected signal integration device.

## *Bacillus subtilis* POSSESSES 2 BY-KINASES (PtkA AND PtkB), ONE COGNATE PHOSPHATASE (PtpZ), AND TWO PUTATIVE PHOSPHOTYROSINE-PROTEIN PHOSPHATASES YfkJ AND YwlE

The first report of proteins being phosphorylated on tyrosine residues by a protein-tyrosine kinase in *Bacillus subtilis* was published by Mijakovic et al. (2003). At the time it was known that some other bacteria, such as *Escherichia coli* and *Streptococcus pneumoniae*, encode proteins that autophosphorylate on tyrosine residues in their C-terminal domains (Vincent et al., 1999; Morona et al., 2000). These autokinases were shown to be implicated in regulating the synthesis of extracellular polysaccharides (Wugeditsch et al., 2001; Bender et al., 2003), and were later named BY-kinases (abbreviation of “bacterial tyrosine kinases”; Grangeasse et al., 2007). Proteins belonging to the BY-kinase family exhibit a surprisingly low degree of sequence homology, with the only conserved features being the catalytic site composed of the Walker A, A’ and B motifs and the autophosphorylation region in their C-termini (Shi et al., 2014b). BY-kinases were identified in *B. subtilis* based on sequence homology with *E. coli* Wzc and *S. pneumoniae* CpsB (Mijakovic et al., 2003). *B. subtilis* possesses two BY-kinases, originally known as YwqD and YveL, which were renamed PtkA and PtkB, respectively (Mijakovic et al., 2005a). PtkB is also known as EpsB (Kearns et al., 2005). One finding that emerged during the initial functional characterization of the *B. subtilis* BY-kinases had a broad impact on the field: BY-kinases not only can autophosphorylate, they can also phosphorylate other cellular proteins, and thus regulate their functions. The first reported BY-kinase substrate was the UDP-glucose dehydrogenase Ugd (YwqF), which was found to be phosphorylated by the *B. subtilis* PtkA (Mijakovic et al., 2003). The Ugd homolog in *E. coli* was also found to be phosphorylated by the PtkA homolog, Wzc (Grangeasse et al., 2003). The second *B. subtilis* BY-kinase, PtkB, was initially not biochemically characterized due to its insolubility (Mijakovic et al., 2003). Genes encoding PtkA and PtkB are adjacent to genes encoding their respective transmembrane activators, TkmA (YwqC) and TkmB (YveK). These proteins have two transmembrane helices with a large extracellular loop between them, and short cytosolic termini, responsible for the interaction with the cytosolic kinase. Structures of some BY-kinases have been resolved, and notably that of CapB from *Staphylococcus aureus* (Olivares-Illana et al., 2008) has provided a number of important structural and functional insights. BY-kinases from *Firmicutes* form octamers, in which the C-terminus of one subunit enters the active site of the adjacent subunit where it gets phosphorylated. Upon trans-autophosphorylation, the BY-kinase octamers dissociate, and this may have important functional consequences, which are discussed in the next section.

*Bacillus subtilis* possesses one polyhistidinol phosphatase-like phosphotyrosine-protein phosphatase, PtpZ (YwqE; Mijakovic et al., 2005a), which dephosphorylates PtkA and its known substrates. PtkA and PtpZ are encoded by the same operon, and thus seem to act in concert. In addition to PtpZ, there are two low molecular weight phosphotyrosine-protein phosphatases in *B. subtilis*: YwlE and YfkJ (Musumeci et al., 2005). YfkJ and YwlE were both suggested to play a role in ethanol stress resistance in *B. subtilis*, but the exact mechanism of this regulation has not been clarified (Musumeci et al., 2005). The crystal structure of YwlE has recently been solved (Xu et al., 2006), and this phosphatase has also been reported to dephosphorylate arginine-phosphorylated CtsR (Fuhrmann et al., 2009). There is overwhelming recent evidence in support of its role in dephosphorylating arginine-phosphorylated proteins (Schmidt et al., 2014).

The last review of the state-of-the art concerning protein-tyrosine phosphorylation in *B. subtilis*, comprising the above mentioned findings, was published almost a decade ago (Mijakovic et al., 2005b). In the perspectives section of that paper it was argued that the next step in the field should be a systematic search for tyrosine-phosphorylated proteins, exploring the possibility that kinases phosphorylate multiple substrates. Another highlighted perspective was the possibility that kinases cross-react with alternative activator proteins and possibly other kinases. As will be discussed in the following sections, these predictions have been to a large extent validated by recent developments in the field.

## LESSONS LEARNED OVER THE LAST DECADE: PtkA IS A HIGHLY CONNECTED REGULATORY DEVICE, PHOSPHORYLATING MANY SUBSTRATES AND REGULATING VARIOUS CELLULAR PROCESSES

Investigations of the *B. subtilis* phosphoproteome started in the era of gel-based proteomics (Lévine et al., 2006), but the first identification of tyrosine-phosphorylated sites came with the gel-free site-specific phosphoproteomics (Macek et al., 2007). Subsequently, a number of phosphoproteomics studies mapped an increasing number of tyrosine-phosphorylated proteins (Eymann et al., 2007; Soufi et al., 2010; Ravikumar et al., 2014). All of the mentioned site-specific phosphoproteome studies, except for Ravikumar et al. (2014), focused on a single experimental point, typically in exponential stage, but in different media. For example, the dataset in Macek et al. (2007) was obtained in LB, Soufi et al. (2010) report the data for the minimal medium with phosphate starvation, etc. The overlap among the reported phosphorylation sites in these different studies is very limited. This can be partly explained by the diversity of the *B. subtilis* phosphoproteome in different stages of growth, which was reported by Ravikumar et al. (2014), highlighting the highly dynamic nature of this reversible modification (Nicolas et al., 2012). But it is also plausible to presume that the reported phosphoproteomes are far from exhaustive due to technical limitations of our current approaches. Ongoing phosphoproteomics studies indicate that the size of the detected bacterial phosphoproteomes is likely to increase dramatically due to new methods for phosphopeptide enrichment. The incompleteness of published phosphoproteomes, and the apparent low level of conservation of phosphorylation sites, seriously limit the performance of available predictors of protein phosphorylation (Iakoucheva et al., 2004; Miller et al., 2009).

**FIGURE 1:**
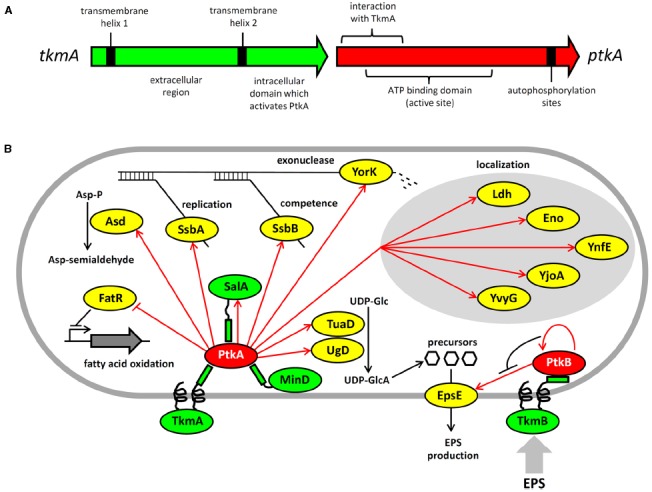
**Architecture and protein substrates of *B. subtilis* BY-kinases. (A)** Schematic structure of the *ptkA* and *tkmA* genes, encoding the kinase and its activator, respectively. **(B)** Network of BY-kinases and their substrates in *B. subtilis.* Proteins are color-coded: BY-kinases are shown in red, kinase activators in green and substrates in yellow. Phosphorylation reactions are indicated with red arrows. Substrate physiological role is indicated next to substrate whose activity is affected by phosphorylation. Substrates whose subcellular localization is affected in the Δ*ptkA* strain are grouped together. The substrate activity is regulated by phosphorylation as follows: Ugd (UDP-glucose dehydrogenase)—activated, Asd (aspartate semialdehyde dehydrogenase)—activated, FatR (repressor of fatty acid oxidation)—inactivated, SsbA and SsbB (ssDNA binding proteins)—activated, YorK (ssDNA exonuclease)—activated, EpsE (glycosyltransferase)—activated.

The phosphoproteomics results immediately invited the question whether PtkA or PtkB could phosphorylate any of these newly-identified tyrosine-phosphorylated proteins. There are no known motifs for substrate recognition by PtkA and PtkB, thus the only way to answer this question was to examine the substrate-kinase relationships experimentally. *In vitro* follow-up studies indicated that PtkA can indeed phosphorylate many of them (Mijakovic et al., 2006; Jers et al., 2010; Figure 1). Nevertheless, tyrosine kinases different from BY-kinases are likely to be present in bacteria. For example, phosphoproteome studies with *Listeria monocytogenes*, a close relative of *B. subtilis*, revealed about a dozen tyrosine-phosphorylated peptides (Misra et al., 2011, 2014). However, no protein resembling BY-kinases is present in this pathogen.

The consequences of PtkA-dependent phosphorylation vary considerably depending on its substrate. Phosphorylation of Ugd led to an increase in its UDP-glucose dehydrogenase activity, and thus production of UDP-glucuronate (Mijakovic et al., 2003; Petranovic et al., 2007). Unphosphorylated tyrosine 70 occupies the active site of Ugd and thus obstructs substrate binding. Phosphorylation displaces tyrosine 70 from the active site and hence activates Ugd function (Petranovic et al., 2009). PtkA also phosphorylates and activates the aspartate dehydrogenase Asd, which converts aspartyl-phosphate to a semi-aldehyde (Jers et al., 2010). A number of substrates phosphorylated and activated by PtkA are involved in single-stranded DNA-metabolism; these include the ssDNA-binding proteins SsbA (Ssb) and SsbB (YwpH; Mijakovic et al., 2006) and the ssDNA-specific exonuclease YorK (Jers et al., 2010). Phosphorylation of these proteins has been linked to the cell cycle and DNA replication phenotype of the Δ*ptkA* mutant (Petranovic et al., 2007), although the details of the underlying mechanism are not clear. PtkA-dependent phosphorylation directly regulates the activity of the protein substrates mentioned above (Ugd, Asd, SsbA, SsbB, YorK), but that is not the only possible outcome of substrate phosphorylation. In case of other tyrosine-phosphorylated proteins revealed by phosphoproteomics: Ldg, Eno, YnfE, YjoA, and YvyG, the PtkA did not control their activity, but seems to be required for their proper cellular localization (Jers et al., 2010). There are also cases where PtkA-dependent phosphorylation had no detectable effect on either activity or localization; such is the case of the peptide transport protein OppA (Jers et al., 2010). PtkA and its cognate phosphatase PtpZ have also been linked with biofilm formation, although their precise roles in this process remain elusive (Kiley and Stanley-Wall, 2010).

While the phosphoproteomics studies continue to provide a growing list of tyrosine-phosphorylated proteins, they do not provide direct evidence for physiological links between these phosphoproteins and kinases and phosphatases responsible for their phosphorylation state. Another global approach, the interactomics, has recently provided important insights in that direction (Shi et al., 2014a). A global two-hybrid interactomics study focused on *B. subtilis* BY-kinases and cognate phosphatases as initial baits revealed a large network of 137 interactions, linking 82 proteins. The capacity of the network to reveal new BY-kinase substrates was demonstrated immediately. The first such characterized substrate was the fatty acid-regulated transcriptional factor FatR, which interacted with TkmA in two hybrid experiments. The transcription regulator was subsequently shown to be phosphorylated by PtkA/TkmA on a tyrosine residue, which plays a key role in FatR interaction with its DNA binding site (Derouiche et al., 2013). Phosphorylation of FatR led to a loss of interaction with DNA, and derepression of its target operon involved in hydroxylation of polyunsaturated fatty acids. Further substrates of PtkA revealed by interactomics were the general recombinase RecA and the cell division protein DivIVA (Shi et al., 2014a), but these have not yet been characterized beyond the *in vitro* phosphorylation studies. The interaction network also provided a number of proteins interacting with both a kinase and a phosphatase, strongly indicating that they may be the substrates of both.

Interestingly, not all BY-kinase interacting proteins turned out to be substrates. The BY-kinases and their respective activators are encoded by a single gene, and thus exist as fusion proteins in Proteobacteria. In Firmicutes, they are encoded by separate genes, and thus theoretically capable of dissociating from one another. Based on this, we have been speculating for some time that BY-kinases may indeed dissociate from their canonical transmembrane activators and interact with other proteins, which may act as alternative activators (Shi et al., 2010). The interaction network in *B. subtilis* provided first evidence for this. We have previously pointed out that PtkA exhibits significant homology with two other Walker motif-containing proteins, MinD and SalA (Mijakovic et al., 2005b). Both MinD and SalA were revealed as PtkA interactants (Shi et al., 2014a). While neither MinD nor SalA possess kinase activity, they both exhibit the capacity to activate PtkA kinase function (Shi et al., 2014a). MinD specifically activates kinase autophosphorylation (Shi et al., 2014a). Our recent results suggest that SalA behaves more like the canonical modulator TkmA, it activates both kinase autophosphorylation and substrate phosphorylation, but the Ptk/SalA complex exhibits different substrate specificity than the PtkA/TkmA complex. This suggests that the purpose of alternative activators for PtkA could be to expand and diversify its substrate pool. Protein localization studies also support the notion that PtkA cycles among different activators in the cell. When *ptkA* and *tkmA* were jointly overexpressed as fluorescent protein fusions, they co-localized at the membrane during the exponential phase, but PtkA left the membrane and became cytosolic in the stationary phase (Jers et al., 2010). In the absence of overexpression, PtkA localized at a single cell pole in a significant fraction of cells in the exponential phase, and this localization was MinD dependent (Shi et al., 2014a). It therefore seems plausible that TkmA, MinD, and SalA represent three alternative anchoring points for PtkA, directing its localization and activity toward different substrates throughout the cell cycle.

## PtkB: AN INSULATED REGULATORY DEVICE, REGULATING ONLY EXOPOLYSACCHARIDE PRODUCTION?

PtkB is encoded by a large *eps* operon involved in production of exopolysaccharides (Elsholz et al., 2014) and biofilm development (Gerwig et al., 2014). PtkB phosphorylates at least one enzyme encoded by the same operon, the glycosyl-transferase EpsE (Elsholz et al., 2014). This phosphorylation mechanism has been described as a positive feedback loop, in which exopolysaccharides act through the transmembrane activator protein TkmB (EpsA, YveK). According to Elsholz et al. (2014), in the presence of exopolysaccharides TkmB prevents autophosphorylation of PtkB and diverts its activity toward the substrate: EpsE. Phosphorylated EpsE is activated, and consequently the production and export of exopolysaccharides increases. This is a very surprising finding, since it has never been reported that autophosphorylation and substrate phosphorylation can be mutually antagonistic in any BY-kinase. In fact, the structural data suggest quite the opposite. The octamer structure of the BY-kinases represents the conformation in which the kinase active sites are inaccessible to substrates (Olivares-Illana et al., 2008). Autophosphorylation triggers octamer dissociation, and theoretically renders the active sites of the kinase accessible to substrates. It will therefore be very interesting to investigate the structural context of PtkB inactivation by autophosphorylation. Whatever the mechanism of PtkB regulation is, the phenotype of Δ*ptkB* and the present studies suggest its involvement uniquely in a single process, the production of extracellular polysaccharides during biofilm development (Elsholz et al., 2014; Gerwig et al., 2014). However, one should not prematurely conclude that PtkB is an insulated regulatory device. Firstly, the role of PtkB is partially complemented by PtkA with respect to biofilm development (Gerwig et al., 2014). Further, interactomics data suggest the ability of Tkm/PtkA and TkmB/PtkB to switch partners, at least at the protein–protein interaction level (Shi et al., 2014a). This suggests a possibility of promiscuity also at the level of substrate phosphorylation. Finally, in the two hybrid screen PtkB interacted directly with MinD, PolA, RpoB, and MutL, which are the key players of housekeeping processes such as division, replication, transcription, and damage repair (Shi et al., 2014a). These interactions in a high confidence network are very likely to have physiological significance, and thus suggest that PtkB may be involved in coordinating exopolysaccharide synthesis with other key cellular processes.

## EMERGING PROPERTIES OF THE BY-KINASE REGULATORY NETWORK IN *B. subtilis*: RAPIDLY EVOLVING BY-KINASES ADOPT NEW SUBSTRATES AND ENGAGE IN INTERACTION WITH OTHER TYPES OF KINASES

A recent study compared the sequences of BY-kinases from all available bacterial genomes, in an attempt to understand their evolutionary history (Shi et al., 2014b). One surprising finding of that study was the apparent hyper-mutability of BY-kinase genes. The synonymous substitution rate in BY-kinase genes was comparable to other bacterial genes. However, the non-synonymous substitution rate in BY-kinase genes was about threefold higher compared to the control. This indicates that BY-kinases accumulate mutations at an increased rate. One direct consequence of this phenomenon is no detectable co-evolution between kinases and their known substrates. This lack of co-evolution means that BY-kinases are promiscuous and can phosphorylate substrate homologs from different bacteria (Shi et al., 2014b). This promiscuity toward substrates thus seems to be “hard-wired” in the evolutionary setup of BY-kinases. Why would bacteria maintain such promiscuous regulatory devices? One possible explanation that was put forward is the maintenance of BY-kinases as rapidly evolving regulators, which can readily adopt new substrates when environmental changes impose selective pressure for rapid evolution of new regulatory modules (Shi et al., 2014b). In that sense, BY-kinases should be seen as sensing/regulatory devices at the forefront of rapid adaptation.

**FIGURE 2:**
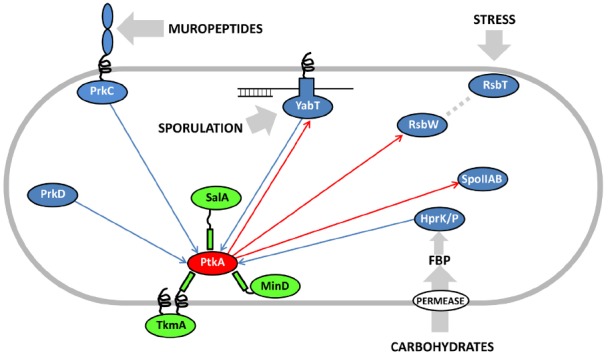
**Cross-phosphorylation among PtkA and serine/threonine kinases from *B. subtilis*.** Proteins are color-coded: BY-kinases are shown in red, kinase activators in green and serine/threonine kinases in blue. If known, activating signals for serine/threonine kinases are shown with gray arrows. Phosphorylation reactions catalyzed by PtkA are shown with red arrows and those catalyzed by the serine/threonine kinases with blue arrows. Adapted from Shi et al. (2014c). FBP stands for fructose-1,6-bisphosphate, which activates the bifunctional HPr kinase/phosphorylase (Mijakovic et al., 2002), a component involved in carbon catabolite repression in firmicutes (Deutscher et al., 2014).

Eukaryal serine/threonine and tyrosine kinases are known to form complex cascades of mutual activation, in which one kinase phosphorylates another (cross-phosphorylation), and which serve as signal integration and amplification devices (Nishida and Gotoh, 1993). No evidence of such cascades existed in bacteria until two very recent studies, in *Mycobacterium tuberculosis* (Baer et al., 2014) and *B. subtilis* (Shi et al., 2014c). Baer et al. (2014) reported cross-phosphorylation among mycobacterial serine/threonine kinases of the Hanks type, while Shi et al. (2014c) detected extensive cross-phosphorylation among serine/threnonine kinases of several distinct families (Hanks type, two-component-like, HprK/P) and the BY-kinases. The BY-kinase PtkA thus engages in extensive cross-phosphorylation interplay with other kinases. In addition to being able to switch activators with PtkB, it also phosphorylates the following serine/threonine kinases: YabT, RsbW and SpoIIAB *in vitro* (Figure 2). In turn, it is phosphorylated by PrkC, PrkD, YabT, and HprK/P. It has not yet been clearly established whether all these cross-phosphorylation events have regulatory roles, but the existing data seem to favor that notion. In the case of phosphorylation of the Hanks-type kinase YabT by PtkA, three phosphorylated tyrosines were detected by mass spectrometry (Shi et al., 2014c). Two of them (Y28 and Y92) are putatively involved in YabT dimerization and the third one (Y254) is in the region essential for DNA binding. DNA binding is the key signal which activates YabT (Bidnenko et al., 2013), and phosphorylation of Y254 is likely to prevent this activation. In the case of PtkA phosphorylation by PrkC, the target residue is S223 (Shi et al., 2014c). The phosphorylated S223 is adjacent to the C-terminal cluster of tyrosines (Y225, Y227, and Y228) which constitute PtkA autophosphorylation sites. As mentioned previously, PtkA autophosphorylation in this region triggers the dissociation of the octameric ring (Olivares-Illana et al., 2008). In agreement with this finding, it has been observed that PtkA autophosphorylation *in vivo* gets strongly enhanced in the Δ*prkC* strain (Ravikumar et al., 2014). Therefore the PrkC-dependent phosphorylation of PtkA could constitute a signal to tune PtkA autophosphorylation levels, and by extension its oligomerization state and access to substrates.

In conclusion, the picture of the regulatory network that emerges around the BY-kinase PtkA is an extremely complex one, with many degrees of connectivity. This kinase phosphorylates and regulates over a dozen cellular substrates, including three other protein kinases (YabT, RsbW, and SpoIIAB). It interacts with three activator proteins: TkmA, MinD, and SalA, but apparently not at the same time, nor the same place. These activators can respectively transmit to PtkA the inputs regarding the presence of exopolysaccharides at the surface, progression of the cell cycle and the exoprotease activity. They also influence the choice of substrates that PtkA can phosphorylate. Finally, PtkA is phosphorylated by four other kinases: PrkC, YabT, HprK/P, and PrkD, which are likely to transmit signals relative to germination, sporulation and sugar availability. The emergent picture is that of a signal integration device that receives a large number of inputs and distributes the outputs via regulation of relevant downstream processes via substrate phosphorylation. From the pleiotropic phenotype of Δ*ptkA* (Petranovic et al., 2007), early on it could be imagined that its cellular role will be complex. Untangling this web of interactions and sorting them out spatially and temporally will require significant additional efforts. PtkB should also not be forgotten in this network reconstruction, as preliminary evidence indicates that it could also be more promiscuous than presently believed. In order to fully explain the roles of PtkA and PtkB in *B. subtilis* physiology, the attention should now be turned to in-depth physiological characterization of all the connected signaling pathways. This effort should be supported by more time-resolved quantitative phosphoproteomics, which should be able to capture the dynamic aspect of these regulatory events. Lessons learned in *B. subtilis* are likely to be of interest for the studies of BY-kinases in pathogenic bacteria, as they are known to play important roles in bacterial virulence (Cozzone, 2009).

### Conflict of Interest Statement

The authors declare that the research was conducted in the absence of any commercial or financial relationships that could be construed as a potential conflict of interest.
